# Association between fat-soluble vitamin co-exposure patterns and blood pressure in people with hypertension: a cross-sectional study

**DOI:** 10.3389/fnut.2024.1502139

**Published:** 2025-01-23

**Authors:** Suming Dai, Ping Wang, Sijia Wang, Hong Chen, Zhixin Cui, Wenhai Lu, Ziyi Zhou, Nan Zhang, Zhuo Wang, Tengfei Lin, Yun Song, Lishun Liu, Xiao Huang, Ping Chen, Genfu Tang, Yong Duan, Hao Zhang, Binyan Wang, Yan Yang, Zezhong Tian

**Affiliations:** ^1^School of Public Health (Shenzhen), Shenzhen Campus of Sun Yat-sen University, Sun Yat-sen University, Shenzhen, China; ^2^Shenzhen Luohu District Chronic Disease Prevention and Treatment Hospital, Shenzhen, China; ^3^Pingdi Public Health Service Center, Shenzhen, China; ^4^Graduate School at Shenzhen, Tsinghua University, Shenzhen, China; ^5^Shenzhen Evergreen Medical Institute, Shenzhen, China; ^6^Department of Cardiology, Peking University First Hospital, Beijing, China; ^7^Key Laboratory of Precision Nutrition and Food Quality, Ministry of Education, Department of Nutrition and Health, College of Food Sciences and Nutritional Engineering, China Agricultural University, Beijing, China; ^8^College of Pharmacy, Jinan University, Guangzhou, China; ^9^Institute of Biomedicine, Anhui Medical University, Hefei, China; ^10^Department of Cardiology, The Second Affiliated Hospital of Nanchang University, Nanchang, China; ^11^School of Heath Administration, Anhui Medical University, Hefei, China; ^12^Yunnan Key Laboratory of Laboratory Medicine, Kunming, China; ^13^Department of Clinical Laboratory, The First Affiliated Hospital of Kunming Medical University, Kunming, China; ^14^National Clinical Research Center for Kidney Disease, State Key Laboratory for Organ Failure Research, Division of Nephrology, Nanfang Hospital, Southern Medical University, Guangzhou, China; ^15^Guangdong Provincial Key Laboratory of Food, Nutrition and Health, Guangzhou, China; ^16^Guangdong Provincial Engineering Laboratory for Nutrition Translation, Guangzhou, China

**Keywords:** fat-soluble vitamins, co-exposure patterns, blood pressure, essential hypertension, cross-sectional study

## Abstract

**Background:**

Existing epidemiological studies investigated the association between a single vitamin and hypertension. However, the potential relationship between the level of circulating multivitamins and blood pressure has not been explored. We aimed to investigate the association between multiple fat-soluble vitamin levels and blood pressure.

**Methods:**

A total of 2052 participants with essential hypertension were sampled nationwide. The plasma concentrations of fat-soluble vitamins (A, E, D, and K) were assessed using liquid chromatography coupled with the mass spectrometry method. Participants were categorized into different co-exposure patterns using the unsupervised K-means clustering method. The multiple linear regression model was used for subsequent analyses.

**Results:**

Participants were classified into two co-exposure patterns of fat-soluble vitamins. The levels of vitamins were relatively low in pattern 1, compared to pattern 2. Participants in pattern 2 had no significantly different blood pressure levels compared to pattern 1. However, the plasma 25-hydroxyvitamin D_3_ (VD_3_) levels were negatively associated with SBP (logarithmic 10 transformed) (*β* = −0.002, 95% CI: −0.004, 0); participants in the fourth *α*-tocopherol quartile had mean SBP levels that were 1.02% (95% CI: 0.43, 1.61%) greater than those in the lowest quartile (*p* for trend <0.01). In addition, no significant relationships were found between plasma VA/VK concentrations and blood pressure.

**Discussion:**

Although no significant association between fat-soluble vitamin co-exposure patterns and blood pressure was found, further analyses could imply that plasma *α*-tocopherol levels may offset the potential protective effect of plasma VD_3_ on blood pressure among hypertensive adults. This provided a novel perspective for exploring the joint effects of fat-soluble vitamins on blood pressure. Further studies are warranted to better understand the implications.

## 1 Introduction

Hypertension poses a great threat to public health worldwide. Globally, almost 1.3 billion adults suffered from hypertension in 2019 (1). Increased blood pressure is considered a major risk factor for premature death. An estimated 8.5 million deaths globally were attributable to high systolic blood pressure in 2015 (2). Owing to the striking prevalence and related significant mortality, the prevention and management of hypertension is imperative. Considering that medication therapy always has some adverse effects (3), modifying lifestyle factors was advocated to improve blood pressure. Apart from improving some traditional risk factors, such as smoking, alcohol consumption, and physical inactivity, the potentiality of nutrients in regulating blood pressure has been given considerable interest recently (4, 5).

Prior epidemiological studies have explored the association between vitamins and blood pressure (6–8). However, the results were equivocal. For instance, individuals with insufficient vitamin D (VD) possibly had a higher risk for hypertension (9), while some intervention trials designed to address the effects of VD supplementation on blood pressure showed inconsistent results (10); some observational studies reported no association between vitamin A (VA) intake and hypertension (11, 12), but an inverse association was observed between dietary VA intake and new-onset hypertension (13).

Most studies mentioned above focused on the relationship between single fat-soluble vitamin instead of multivitamin exposure and blood pressure. However, recent studies demonstrated that some vitamins may exert their blood pressure regulatory functions by interacting with other vitamins (14, 15). Moreover, the assessment of some vitamin exposure levels via estimating the vitamin contents of diets and supplements in numerous previous studies is far from accurate, because vitamin loss may occur during food storage, processing, or cooking and the absorption of vitamins varies among different populations (16–18). Using metabolomics methods such as liquid chromatography coupled with mass spectrometry (LC–MS) to detect circulating vitamins and/or their direct metabolites could be more reliable for vitamin status assessment (19, 20). Up to now, there has been no study exploring the relationship between circulating multivitamin status and blood pressure in hypertensive adults.

Clustering methods, as a powerful unsupervised machine algorithm, have been used in the nutrition field to determine dietary patterns and in the environmental health field to identify mixed pollutant exposure (21, 22). It is implied that the classification of individuals based on their vitamin exposure patterns through clustering methods could be effective in exploring the association between vitamin co-exposure and blood pressure.

Accordingly, the present study was conducted primarily to elucidate the associations between circulating concentrations of multivitamins (vitamins A, D, E, and K) and blood pressure among hypertensive adults.

## 2 Methods

### 2.1 Study design and participants

A multicenter epidemiological study, initiated in February 2017 with ongoing enrollment, was conducted to identify, register, and educate the hypertensive population in China. The inclusion criteria for study participants contained the following: (1) systolic/diastolic blood pressure ≥ 140/90 mmHg or taking antihypertensive drugs (23); and (2) voluntarily participating and signing the written informed consent. The exclusion criteria contained the following: (1) suffering from serious mental disorders or being unable to express themselves; and (2) having other obvious abnormal physical signs, laboratory detecting results, or clinical diseases, unable to participate. The study complied with the Declaration of Helsinki and was approved by the Ethics Committee of Peking University First Hospital, Beijing, China (Ethics code: 20161231). During the enrollment, individuals were informed of the study protocol and then decided by themselves whether to participate in the study. It was entirely voluntary. The informed consent would be signed only if they wanted to participate.

Two subsamples from this ongoing study without duplication were the participants in the current study. Briefly, stratified by province, 800 individuals enrolled from June to August 2017 were firstly selected from 9 provinces (Beijing, Hebei, Liaoning, Gansu, Guangxi, Hunan, Jiangsu, Sichuan, and Jiangxi) at random. Then another 1,543 participants enrolled from February 2017 to May 2018 were randomly selected from 14 provinces (Anhui, Ningxia, Heilongjiang, Shandong, Yunnan, and the 9 provinces in the first sampling). Finally, after combining two subsamples and excluding outliers, a total of 2052 participants were included (Supplementary Figure S1).

### 2.2 Basic characteristics of participants

Participants’ anthropometric, sociodemographic, lifestyle, and comorbid factors were collected. Calibrated instruments were used to measure height and weight to an accuracy of 0.1 cm and 0.1 kg, respectively. Body mass index (BMI) was computed by dividing weight (kg) by height squared (m^2^).

Participants’ age, sex, ethnicity, northern or southern region, marital status, education level, smoking status, alcohol use, physical labor intensity, living standard, nervousness, and use of anti-hypertension medications were obtained by questionnaire. The marital status of participants was divided into five categories: married, widowed, divorced or separated, never married, and others. Level of education was reported on a 9-category scale and was further classified into three broad categories: lower levels (primary school or less), medium levels (general intermediate education or intermediate vocational education), and higher levels (general secondary education, higher vocational education, bachelor, master or higher).

Smoking and drinking status were categorized as never, former, and current tobacco or alcohol users. Participants reported the start time and the amount of smoking or alcohol consumed. Physical labor intensity was categorized as mild, moderate, and heavy levels. Nervousness was self-reported as mild, moderate, and severe degrees. The living standard was classified into three grades: poor, average, and good. In addition, the medical history of hypertension, diabetes, dyslipidemia, stroke, and coronary heart disease was asked of participants. The use of antihypertensive drugs and multivitamin supplements was investigated.

### 2.3 Measurement of blood pressure

Systolic and diastolic blood pressures were measured in the upper arm after 15 min of seated rest, using an electronic sphygmomanometer (Yuwell brand). The right brachial artery blood pressure should be measured at least three times, with an interval of 3–5 min between each measurement. The difference between each measurement should be less than 10 mmHg (23). We calculate the mean blood pressure by several measurements.

### 2.4 Measurement of vitamins

Blood samples were collected from all participants. Plasma was separated within 30 min after venous blood collection and stored at −80°C temperature until detection. Plasma concentrations of VA (retinol), VD (25-hydroxyvitamin D_3_ [25(OH)D_3_]), VE (*α*-tocopherol), and VK levels were measured using liquid chromatograph-mass spectrometer (LC–MS) method in Beijing DIAN Medical Diagnostics Laboratory (24, 25). The corresponding standard sample was used as a reference. For quality control, duplicate samples were randomly placed in the detected samples, and the coefficients of variation for them from the same batch and different batches were calculated. During the detection process, the personnel were unaware of the grouping status of the samples.

### 2.5 Statistical methods

The basic characteristics of participants were described as median and interquartile range for skewed continuous variables and proportions for categorical variables. The Wilcoxon rank-sum test and chi-square test were used to compare the differences of basic characteristics between the two subsamples.

Participants’ multivitamin co-exposure patterns were determined through the K-means clustering method. Average silhouette width, Gap Statistic, “NbClust” package in R, and biological interpretability were considered to help determine the appropriate number of clusters (26). Afterward, we included the co-exposure pattern in the multiple linear regression model to explore its association with blood pressure. We further used stratified analyses and interaction tests to explore the possible modifiers of the association between co-exposure patterns and blood pressure.

Additionally, we also used simple and multiple linear regression analyses to investigate the association between each vitamin and blood pressure. As for multiple linear regression models, model 1 was adjusted for age, sex, and BMI. Model 2 was further adjusted for region, physical labor intensity, living standard, nervousness, education levels, and smoking and drinking status. Model 3 was additionally adjusted for the family history of hypertension, stroke, coronary heart disease, comorbidity (dyslipidemia and diabetes), and the use of anti-hypertension drugs. Furthermore, restricted cubic spline regression analysis was used to explore the potential non-linear association between each vitamin and blood pressure.

All statistical analyses were performed using R software, version 4.1.2. The two-sided *p*-value of <0.05 was considered statistically significant in all analyses.

## 3 Results

### 3.1 Basic characteristics of study participants

The sociodemographic, anthropometric, lifestyle, and comorbid characteristics of participants are presented in Table 1. Among the 2052 included participants, the mean age was 63.8 (SD, 13.2) years, and 52.9% of participants were male. The median SBP and DBP of the participants were 144 (133, 154) mmHg and 88 (80, 95) mmHg, respectively. More than half of the participants (59.2%) had a higher BMI (≥24 kg/m^2^), and 1,124 (54.8%) participants reported a family history of hypertension. According to the survey, 0.3% of the total participants claimed the supplementation of multivitamins. There were 1,505 (73.3%) individuals using anti-hypertension drugs. The mean concentrations of plasma retinol and 25(OH)D3 were 0.53 ± 0.17 g/mL and 19.51 ± 8.57 μg /L, respectively. The median concentrations of plasma *α*-tocopherol and vitamin K were 10.27 (8.33, 12.50) g/mL and 0.94 (0.53, 1.62) μg /L, respectively.

**Table 1:** Basic characteristics of all participants in this study.

Characteristics	Sample 1 (*n* = 715)	Sample 2 (*n* = 1,337)	Total (*n* = 2052)	*p*-value*
*n* (%)				
Male	418 (58.5%)	667 (49.9%)	1,085 (52.9%)	<0.001*
Age (years)				
20–39	25 (3.50%)	40 (2.99%)	65 (3.17%)	0.783
40–59	244 (34.1%)	468 (35.0%)	712 (34.7%)	
≥60	446 (62.4%)	829 (62.0%)	1,275 (62.1%)	
Ethnicity				
Han	589 (93.5%)	1,127 (94.1%)	1716 (93.9%)	0.697
Other ethnic minorities	41 (6.51%)	71 (5.93%)	112 (6.13%)	
Region				
North	399 (55.8%)	803 (60.1%)	1,202 (58.6%)	0.069
South	316 (44.2%)	534 (39.9%)	850 (41.4%)	
Education				
Primary school or less	302 (42.2%)	560 (41.9%)	862 (42.0%)	0.052
General intermediate education	232 (32.4%)	379 (28.3%)	611 (29.8%)	
General secondary education or higher	181 (25.3%)	398 (29.8%)	579 (28.2%)	
Marital status				
Married	600 (83.9%)	1,086 (81.2%)	1,686 (82.2%)	0.643
Widowed	105 (14.7%)	226 (16.9%)	331 (16.1%)	
Divorced or separated	4 (0.56%)	12 (0.90%)	16 (0.78%)	
Never married	5 (0.70%)	10 (0.75%)	15 (0.73%)	
Others	1 (0.14%)	3 (0.22%)	4 (0.19%)	
BMI				
<18.5 kg/m^2^	21 (2.94%)	35 (2.62%)	56 (2.73%)	0.704
18.5–23.9 kg/m^2^	283 (39.6%)	498 (37.2%)	781 (38.1%)	
24.0–27.9 kg/m^2^	328 (45.9%)	644 (48.2%)	972 (47.4%)	
≥28.0 kg/m^2^	83 (11.6%)	160 (12.0%)	243 (11.8%)	
Smoking				
Never	480 (67.1%)	964 (72.1%)	1,444 (70.4%)	0.003*
Former	69 (9.65%)	146 (10.9%)	215 (10.5%)	
Current	166 (23.2%)	227 (17.0%)	393 (19.2%)	
Alcohol drinking				
Never	530 (74.1%)	1,007 (75.3%)	1,537 (74.9%)	0.096
Former	44 (6.15%)	107 (8.00%)	151 (7.36%)	
Current	141 (19.7%)	223 (16.7%)	364 (17.7%)	
Physical labor intensity				
Mild	498 (69.7%)	937 (70.1%)	1,435 (69.9%)	0.484
Moderate	188 (26.3%)	332 (24.8%)	520 (25.3%)	
Heavy	29 (4.06%)	68 (5.09%)	97 (4.73%)	
Living standard				
Poor	23 (3.22%)	59 (4.41%)	82 (4.00%)	0.366
Average	486 (68.0%)	883 (66.0%)	1,369 (66.7%)	
Good	206 (28.8%)	395 (29.5%)	601 (29.3%)	
Nervousness				
Mild	515 (72.0%)	1,006 (75.2%)	1,521 (74.1%)	<0.001*
Moderate	176 (24.6%)	246 (18.4%)	422 (20.6%)	
Severe	24 (3.36%)	85 (6.36%)	109 (5.31%)	
Dyslipidemia				
No	581 (81.3%)	1,002 (74.9%)	1,583 (77.1%)	0.001*
Yes	134 (18.7%)	335 (25.1%)	469 (22.9%)	
Diabetes				
No	592 (82.8%)	1,113 (83.2%)	1705 (83.1%)	0.844
Yes	123 (17.2%)	224 (16.8%)	347 (16.9%)	
Family history of hypertension				
No	292 (40.8%)	560 (41.9%)	852 (41.5%)	0.899
Yes	396 (55.4%)	728 (54.5%)	1,124 (54.8%)	
Unknown	27 (3.78%)	49 (3.66%)	76 (3.70%)	
Family history of stroke				
No	556 (77.8%)	1,095 (81.9%)	1,651 (80.5%)	0.072
Yes	135 (18.9%)	209 (15.6%)	344 (16.8%)	
Unknown	24 (3.36%)	33 (2.47%)	57 (2.78%)	
Family history of CHD				
No	624 (87.3%)	1,146 (85.7%)	1770 (86.3%)	0.418
Yes	65 (9.09%)	146 (10.9%)	211 (10.3%)	
Unknown	26 (3.64%)	45 (3.37%)	71 (3.46%)	
Median (P_25_, P_75_)				
SBP (mmHg)	145 (135, 155)	143 (133, 154)	144 (133, 154)	0.071
DBP (mmHg)	89.0 (81.0, 95.0)	88.0 (79.0, 95.0)	88.0 (80.0, 95.0)	0.049
VA (g/mL)	0.52 (0.43, 0.64)	0.50 (0.40, 0.61)	0.51 (0.41, 0.62)	<0.001*
VD (μg /L)	19.7 (14.2, 25.6)	18.1 (12.7, 24.6)	18.7 (13.2, 25.0)	0.002*
VE (g/mL)	9.94 (8.07, 12.1)	10.4 (8.50, 12.8)	10.3 (8.33, 12.5)	<0.001*
VK (μg/L)	0.84 (0.48, 1.45)	1.01 (0.56, 1.71)	0.94 (0.53, 1.62)	<0.001*

VA, vitamin A; VD, vitamin D; VE, vitamin E; VK, vitamin K； BMI, body mass index; CHD, coronary heart disease; P_25_, 25th percentile; P_75_, 75th percentile.

*p*-values were calculated by chi-square test and Wilcoxon rank-sum test for categorical and continuous variables, respectively. The symbol * represents statistically significant results.

### 3.2 Multiple fat-soluble vitamin co-exposure patterns of participants

The heatmap elucidating the pairwise correlation among the four studied vitamins is shown in Supplementary Figure S2. Correlation coefficients between any two studied vitamins ranged from 0.08 to 0.45. The significantly positive correlation was displayed between every two vitamins.

**Figure 1:**
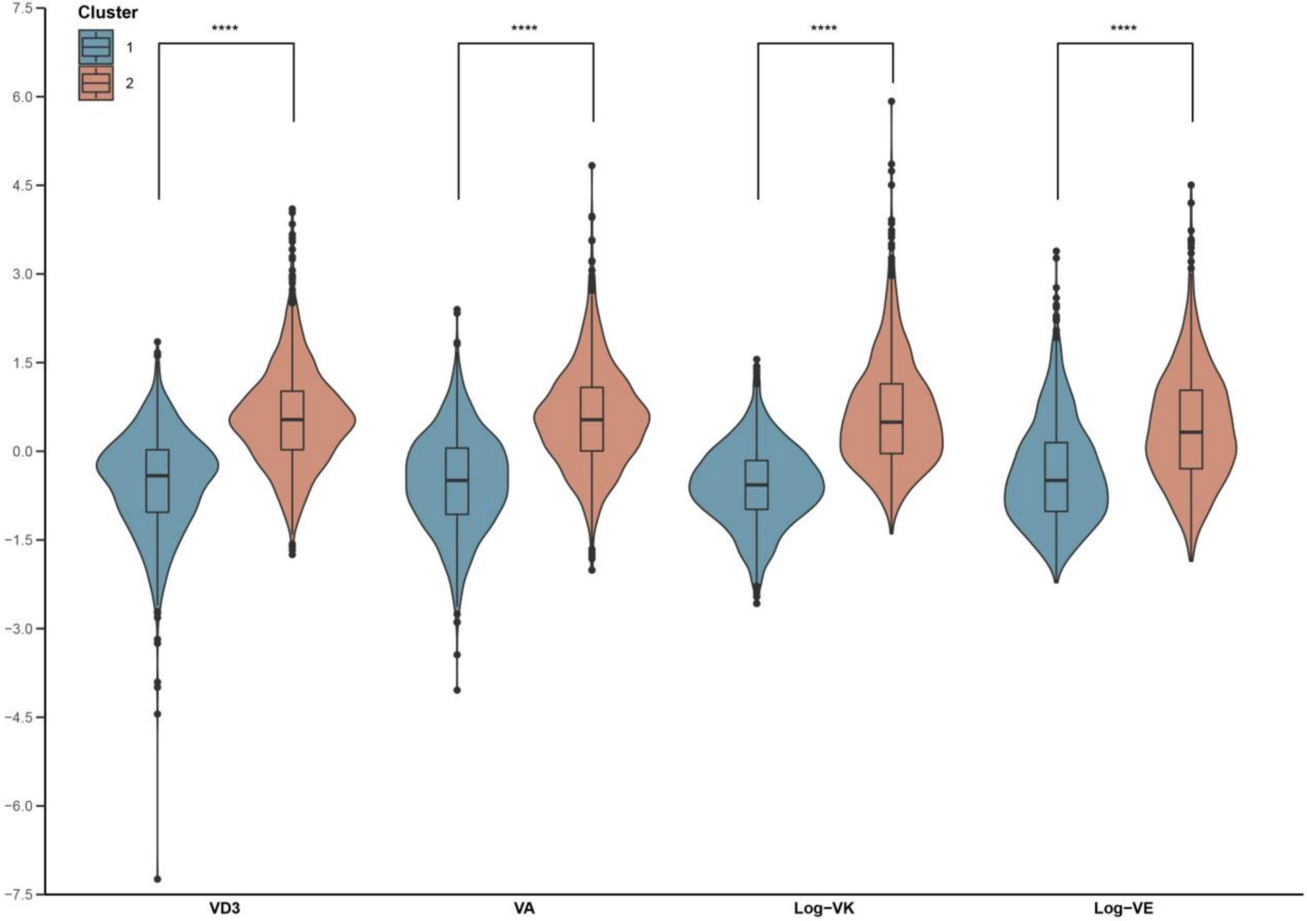
Violin plot of plasma vitamin concentrations grouped by the two clusters. The x-axis indicated the four vitamins and the different two colors represented the two clusters. The y-axis indicates plasma vitamin concentrations. VA, vitamin A; VD, vitamin D; VE, vitamin E; VK, vitamin K. Log indicated logarithmic 10 transformation. The symbol **** represents statistically significant results.

The values of circulating vitamin levels were standardized to eliminate the dimensional difference. The distributions of standardized values are shown in Supplementary Table S1. Based on standardized values, the 2052 participants were classified into two clusters, determined by both statistical results and biological interpretability. The centers of the two clusters are shown in Supplementary Table S2. With respect to the two clusters, we designated the ‘low-level exposure group’ to cluster 1, considering the values of plasma vitamin concentrations close to their 25th percentiles, ‘high-level exposure group’ to cluster 2 for the reason that the values of plasma vitamin concentrations close to their 75th percentiles. The levels of all studied vitamins were relatively low in cluster 1, while the levels were relatively high in cluster 2. Participants in clusters 1 and 2 occupied approximately the same proportions of the total sample. The differences between the studied vitamin concentrations of the two clusters were visualized through a violin plot with a boxplot (Figure 1).

After clustering analysis, we further described the basic characteristics of study participants according to the two clusters, as shown in Supplementary Table S3. There existed significant differences in almost all basic characteristics between the two clusters. In brief, those with lower levels of studied vitamin co-exposure were more likely to be ≥60 years old (71.4%), northerners (68.5%), never drinking (79.4%), and have lower education levels, and less likely to be overweight and have dyslipidemia and family histories of chronic diseases.

### 3.3 Relationship of multiple fat-soluble vitamin co-exposure and blood pressure

Considering the values of blood pressure were skewed, we log-transformed the data. After adjusting for age, sex, and BMI, participants in the high-level exposure group had no significantly different SBP levels compared to the reference group (low-level exposure group). After further adjustment for potential confounders (models 2 and 3), the results remained unchanged. Similarly, no significant association was observed between studied vitamin co-exposure patterns and DBP (Table 2).

**Table 2:** Covariates adjusted *β* coefficient (95% CI) of multivitamin co-exposure patterns in association with blood pressure.

Variable	Model 1		Model 2		Model 3	
	*β* coefficient (95% CI)	*p*-value	*β* coefficient (95% CI)	*p*-value	*β* coefficient (95% CI)	*p*-value
Systolic blood pressure (logarithm 10 transform)						
Cluster 1	Ref		Ref		Ref	
Cluster 2	0 (−0.004, 0.004)	0.988	0.001 (−0.003, 0.005)	0.689	0.001 (−0.003, 0.005)	0.61
Diastolic blood pressure (logarithm 10 transform)						
Cluster 1	Ref		Ref		Ref	
Cluster 2	0.001 (−0.004, 0.006)	0.725	0.001 (−0.004, 0.006)	0.588	0.002 (−0.003, 0.007)	0.36

Model 1 was adjusted for age, sex, and BMI.

Model 2 was adjusted for age, sex, BMI, region, physical labor intensity, living standard, nervousness, education level, smoking status, and alcohol-drinking status.

Model 3 was further adjusted for the family history of chronic diseases (hypertension, stroke, and coronary heart disease), the comorbidity (dyslipidemia and diabetes), and the use of anti-hypertension drugs.

We performed stratified analyses according to potential factors to estimate potential modifying effects (Supplementary Table S4). The associations of multivitamin co-exposure patterns and DBP were stronger among participants <65 years old (*p* for interaction = 0.05), with general intermediate education (*p* for interaction = 0.004) and currently drinking (*p* for interaction = 0.031). There was no other significant interaction found between multivitamin co-exposure patterns and DBP/SBP (all *p* for interaction ≥0.05).

### 3.4 Association of each studied vitamin and blood pressure

The preliminary exploration for the association of each studied vitamin and blood pressure (logarithmic 10 transformed) was made through univariate linear regression. The results were shown by scatter plots and fitted lines with the 95% CIs (Supplementary Figure S3). All four vitamins were significantly associated with DBP, but not SBP. In the multiple linear regression model 3 (Table 3), the plasma VD levels were in a significantly negative association with SBP (*β* coefficient = −0.002, 95% CI: −0.004, 0). A similar association could be found between plasma VD levels and DBP, but was insignificant. In contrast, after adjusting for age, sex, and BMI, higher SBP levels were observed with per SD increment in plasma VE concentrations (*β* coefficient = 0.002, 95% CI: 0, 0.004). After further adjustment (models 2 and 3), the results remained significant (*β* coefficient = 0.003, 95% CI: 0.001, 0.005 in model 2; *β* coefficient = 0.004, 95% CI: 0.002, 0.006 in model 3). The concordant result was obtained for the association of plasma VE concentration and DBP in model 3. In addition, no significant relationships were found between plasma VA/VK concentrations and SBP/DBP.

**Table 3:** Multivariable associations of fat-soluble vitamin concentrations and blood pressure.

Variables		Log-SBP		Log-DBP	
		*β* coefficient (95% CI)	*p*-value	*β* coefficient (95% CI)	*p*-value
VA	Model 1	0 (−0.002, 0.003)	0.729	−0.001 (−0.003, 0.002)	0.511
	Model 2	0.001 (−0.001, 0.003)	0.414	−0.001 (−0.003, 0.002)	0.496
	Model 3	0.001 (−0.001, 0.004)	0.18	0 (−0.002, 0.002)	0.946
VD	Model 1	−0.001 (−0.003, 0.001)	0.248	0 (−0.003, 0.002)	0.796
	Model 2	−0.001 (−0.004, 0.001)	0.216	0 (−0.003, 0.002)	0.956
	Model 3	**−0.002 (−0.004, 0)**	**0.053**	−0.001 (−0.003, 0.002)	0.535
Log-VE	Model 1	**0.002 (0, 0.004)**	**0.04**	0.001 (−0.001, 0.003)	0.346
	Model 2	**0.003 (0.001, 0.005)**	**0.012**	0.001 (−0.001, 0.004)	0.238
	Model 3	**0.004 (0.002, 0.006)**	**0.001**	**0.003 (0, 0.005)**	**0.028**
Log-VK	Model 1	0 (−0.002, 0.002)	0.781	0.001 (−0.002, 0.003)	0.562
	Model 2	0 (−0.002, 0.003)	0.693	0.001 (−0.002, 0.003)	0.612
	Model 3	0.001 (−0.001, 0.003)	0.292	0.002 (−0.001, 0.004)	0.202

Log indicated logarithmic 10 transformation. Data were presented as *β* coefficient (95% CI) per SD of the vitamin.

Model 1 was adjusted for age, sex and BMI.

Model 2 was additionally adjusted for age, sex, BMI, region, physical labor intensity, living standard, nervousness, education level, smoking status, alcohol drinking status.

Model 3 was further adjusted for the family history of chronic diseases (hypertension, stroke and coronary heart disease), the comorbidity (dyslipidemia and diabetes) and the use of anti-hypertension drug.

Bold values indicated statistical significance (*p* < 0.05).

**Figure 2:**
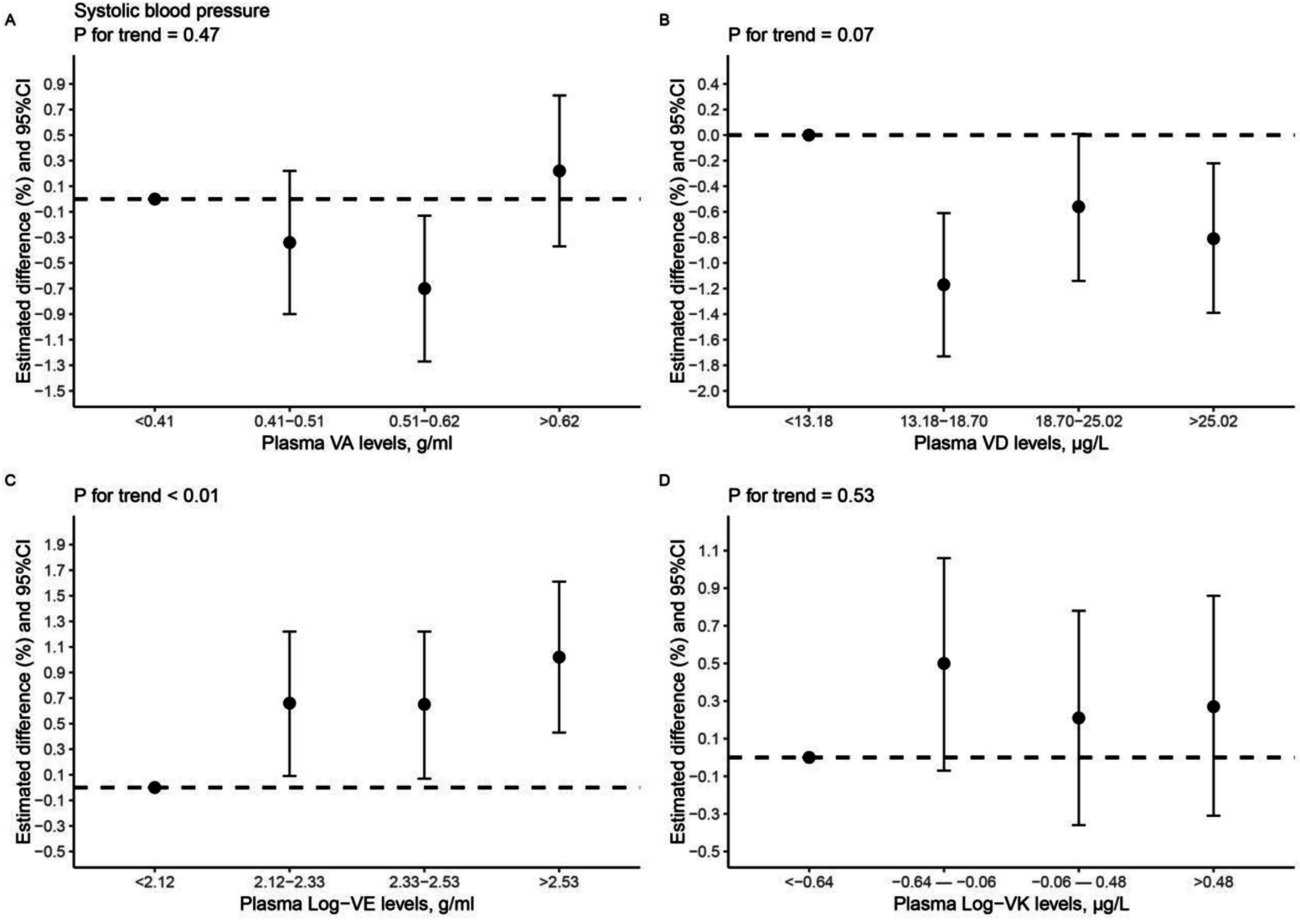
Estimated difference (%) and 95% CI in systolic blood pressure with *p* for trend for each interquartile in plasma vitamin concentrations. **(A)** VA and SBP; **(B)** VD and SBP; **(C)** VE and SBP; **(D)** VK and SBP. VA, vitamin A; VD, vitamin D; VE, vitamin E; VK, vitamin K; SBP, systolic blood pressure. Log indicated logarithmic 10 transformation.

In addition, similar results were obtained when we categorized vitamin variables into quartiles. In the full-adjusted model, participants in the fourth VE quartile had mean SBP levels that were 1.02% (95% CI: 0.43, 1.61%) greater than those in the lowest VE quartile (*p* for trend <0.01, Figure 2). As for DBP, higher plasma levels of VE were associated with elevated mean DBP levels, albeit this trend was not statistically significant (*p* for trend = 0.07, Supplementary Figure S4). On the contrary, individuals in the higher VD quartiles had lower mean SBP levels compared to those in the first VD quartile, although the significance was attenuated in the third VD quartile and the trend was insignificant (*p* for trend = 0.07, Figure 2).

Dose–response curves for the relationship between each studied vitamin and SBP/DBP are shown in Supplementary Figure S5, which presented similar trends to the results aforementioned. No significant non-linear associations were between each studied vitamin and SBP/DBP (all *p* for non-linearity ≥0.05).

## 4 Discussion

In this current study, we evaluated joint effects on blood pressure of circulating multiple fat-soluble vitamins in nationally representative Chinese adults. There was no significant relationship between multiple fat-soluble vitamins and blood pressure. Further analyses on the separate associations of each vitamin and blood pressure showed that there was an inverse relationship between plasma VD level and SBP, but a positive association between plasma VE and SBP.

### 4.1 Multiple fat-soluble vitamin co-exposure patterns and blood pressure

People are usually exposed to multiple vitamins from diverse foods and natural environments in their daily lives. In our study, we defined two vitamin co-exposure patterns by distinct plasma vitamin concentration profiles. The k-means clustering method derived two co-exposure patterns that yielded simple structure and great interpretability, as follows: low-level fat-soluble vitamins exposure and high-level fat-soluble vitamins exposure. In line with the present study, this method was also used in previous studies to explore the relationship between multiple nutrients and various chronic diseases (27–29), which served as evidence for the practicability of our study method.

Few studies have explored the association between multiple fat-soluble vitamins and hypertension. A recent cross-sectional study demonstrated that the factor analysis derived nutrient pattern of fats and fat-soluble vitamins was significant in relation to the prevalence of high blood pressure (29), which somewhat stood by our results of stratified analyses. Additionally, a few randomized controlled trials (RCTs) focused on the efficacy of multivitamin supplementation on blood pressure. One meta-analysis pooling the results of relevant RCTs showed that the lowering effect of multivitamin supplementation on SBP was significant in hypertensive patients, but not on DBP (30). However, the sample size for the hypertension subgroups was only 58. In addition, the above studies only explored the relationship between vitamin intake and blood pressure from the perspective of dietary evaluation or supplements. Previous studies have found that the circulating levels of fat-soluble vitamins can reflect the long-term exposure level of vitamins in the body (31, 32). To our knowledge, the simultaneous effects of circulating multiple vitamin concentrations on blood pressure have not been investigated among hypertensive adults yet. Our study included more hypertensive participants to investigate the association between multiple fat-soluble vitamins in circulation and blood pressure. Overall, there was no significant association between multiple fat-soluble vitamin exposure levels and blood pressure. This may be due to the different relationships between different fat-soluble vitamins and hypertension. Our further analysis also found that there was an inverse relationship between plasma VD levels and SBP, but a positive correlation between plasma VE levels and SBP.

### 4.2 The plasma 25(OH)D_3_ levels and blood pressure

Among the four studied vitamins, we observed an inverse association with SBP for plasma 25(OH)D_3_ concentrations. The result was in line with evidence from similar observational studies relating to VD status and blood pressure. The negative correlation between circulating 25(OH)D levels and SBP was also found in the Chinese rural population (33), albeit the study subjects from a certain province were less than those in this nationwide study. Consistent with our finding, another study among US hypertensive adults unraveled that lower circulating 25(OH)D levels were associated with higher SBP/DBP by 0.5/2.4 mmHg (34). Potential mechanisms underlying the relationship between VD and blood pressure have been investigated via experimental studies, including regulating the renin–angiotensin–aldosterone system (RAAS), activating the nuclear VD receptor highly expressed in the vascular smooth muscle endothelium and cardiomyocytes, and attenuating inflammation through direct action with nuclear factor kappa beta (35, 36).

However, data emerging from intervention trials to evaluate the impact of VD supplementation on hypertension were inconclusive. The recent umbrella review demonstrated that the association between VD concentration and hypertension was only statistically significant in meta-analyses of observational studies or Mendelian randomization studies. In contrast, meta-analyses of RCTs reported marginally significant or no effects of VD supplementation on the prevention or improvement of hypertension (37). This discrepancy was likely linked to the inducer of endogenous VD and population-specific study characteristics. Cutaneous VD_3_ is the predominant source of systemic VD instead of dietary intake or VD supplements. The synthesis of VD_3_ is driven by UV-B radiation from sunlight, which could be affected by many long-term natural factors including latitude, season, or atmosphere construction (38, 39). Accordingly, the limited duration of VD supplementation could not compensate for the long-term VD_3_ insufficiency. In addition, numerous individual factors containing skin pigmentation, age, and obesity may also influence the solar VD_3_ synthesis (38, 40, 41). As a result, specific populations appear to be prone to a more effective response to VD supplementation in terms of regulatory effects on blood pressure.

### 4.3 The plasma *α*-tocopherol levels and blood pressure

A recent meta-analysis of RCTs in general populations found no significant lowering effects of VE supplementation on both SBP and DBP yet (42). However, there existed inconsistent results which showed that 200 IU/day VE supplement for 27 weeks could significantly decrease SBP and DBP in mild hypertensive adults (43). This discrepancy could be partially owing to different forms of VE. Vitamin E, as a powerful antioxidant, has eight isomers and among them, *α*-tocopherol is the most biologically active one (44). Most trials did not investigate the α-tocopherol levels as did in this current study but explored the effects of VE supplements on blood pressure directly or combined with other antioxidants (45). In addition, the multiplicity of epidemiological studies explored the effects of tocotrienol supplementation or *γ*-tocopherol mainly from dietary VE on blood pressure (46).

In contrast, we investigated the effects of circulating *α*-tocopherol on blood pressure. There was a significant trend toward the positive relationship between achieved VE levels and blood pressure. Epidemiological evidence for the relationship between VE and blood pressure was limited and controversial (44). Concordant with our results, one cross-sectional study in the Korean general population also reported that blood pressure was positively associated with serum α-tocopherol levels (47). The above suggests that α-tocopherol may mask the protective effect of plasma 25-hydroxyvitamin D3 on blood pressure.

### 4.4 Strengths and limitations

Our study comes with several strengths. We for the first time assessed the association of multivitamin co-exposure levels and blood pressure among hypertensive adults. The co-exposure patterns of multiple vitamins were assessed via the unsupervised K-means clustering method. Due to a lack of clinical criteria, the machine learning method helps to classify the circulating vitamin levels of hypertensive adults. In addition, we assessed the vitamin status by measuring the distinct active products of vitamins, which have been validated to be reliable markers for vitamins. The majority of the prior studies evaluated vitamin intake by dietary questionnaires, which may have unavoidable limitations.

Nevertheless, our study has also some limitations that should be noted. First, the present cross-sectional study could not determine the causation between the plasma vitamin levels and blood pressure due to the observational design. Second, the confounding effects from unmeasured or unknown variables may not be excluded, albeit we have adjusted many confounders in our models. Finally, the current study only included Chinese hypertensive adults, it is unsure about the multivitamin co-exposure patterns among other population and their relationships to blood pressure.

## 5 Conclusion

Although we did not find a significant association between fat-soluble vitamin co-exposure and blood pressure, higher plasma VD levels were associated with reduced SBP among study participants. There was a significant increase in blood pressure with the rise of plasma VE levels. Our findings provided a novel perspective for exploring the joint effects of fat-soluble vitamins on blood pressure. Further studies are warranted to better understand the implications.

## Data Availability

The raw data supporting the conclusions of this article will be made available by the authors, without undue reservation.
