# Supplementary material for: Association between fat-soluble vitamin co-exposure patterns and blood pressure in people with hypertension: a cross-sectional study
Source: Front Nutr. 2025 Jan 23;11:1502139. doi: 10.3389/fnut.2024.1502139 (PMC11801223; doi:10.3389/fnut.2024.1502139)
Supplement: Supplementary file 1 [file Table_1.docx]

**Table S1.** Distributions of standardised circulating levels of the studied vitamins.

| Variables | Min | 25^th^ percentile | Median | 75^th^ percentile | Max |
| --- | --- | --- | --- | --- | --- |
| VA | -2.580 | -0.690 | -0.100 | 0.560 | 5.940 |
| VD3 | -2.200 | -0.740 | -0.080 | 0.648 | 4.450 |
| Log-VE | -7.220 | -0.610 | 0.015 | 0.600 | 4.090 |
| Log-VK | -3.530 | -0.680 | 0.000 | 0.640 | 4.830 |

VA, vitamin A; VD, vitamin D; VE, vitamin E; VK, vitamin K.

Log indicated logarithmic 10 transformation.

**Table S2.** Centres of the two clusters of the four lipid-soluble vitamins­ with prior log-transformation and standardisation.

| Variables | VA | VD | Log-VE | Log-VK |
| --- | --- | --- | --- | --- |
| Cluster 1 (Low-level exposure) | -0.568 | -0.381 | -0.513 | -0.509 |
| Cluster 2 (High-level exposure) | 0.634 | 0.421 | 0.573 | 0.569 |

VA, vitamin A; VD, vitamin D; VE, vitamin E; VK, vitamin K.

Log indicated logarithmic 10 transformation.

The K-means clustering method clustered 2052 participants into two groups based on data points of the 4 lipid-soluble vitamins. With respect to the two clusters, we designated ‘low-level exposure group’ to cluster 1, considering the values of plasma vitamin concentrations close to their 25^th^ percentiles, ‘high-level exposure group’ to cluster 2 for the reason that the values of plasma vitamin concentrations close to their 75^th^ percentiles

**Table S3.** Baseline characteristics of study participants grouped by the two clusters based on the studied vitamin concentrations of all participants.

| Characteristics | Cluster 1^a^ | Cluster 2^b^ | *P* value* |
| --- | --- | --- | --- |
| **n (%)** | 1085 (52.9%) | 967 (47.1%) |  |
| Male | 546 (50.3%) | 539 (55.7%) | 0.016* |
| Age (years) |  |  |  |
| 20-39 | 22 (2.03%) | 43 (4.45%) | <0.001* |
| 40-59 | 288 (26.5%) | 424 (43.8%) |  |
| ≥60 | 775 (71.4%) | 500 (51.7%) |  |
| Ethnicity |  |  |  |
| Han | 926 (94.4%) | 790 (93.3%) | 0.368 |
| Other ethnic minorities | 55 (5.61%) | 57 (6.73%) |  |
| Region |  |  |  |
| North | 743 (68.5%) | 459 (47.5%) | <0.001* |
| South | 342 (31.5%) | 508 (52.5%) |  |
| Education |  |  |  |
| Primary school or less | 532 (49.0%) | 330 (34.1%) | <0.001* |
| General intermediate education | 302 (27.8%) | 309 (32.0%) |  |
| General secondary education or higher | 251 (23.1%) | 328 (33.9%) |  |
| Marital status |  |  |  |
| Married | 845 (77.9%) | 841 (87.0%) | <0.001* |
| Widowed | 223 (20.6%) | 108 (11.2%) |  |
| Divorced or separated | 7 (0.65%) | 9 (0.93%) |  |
| Never married | 6 (0.55%) | 9 (0.93%) |  |
| Others | 4 (0.37%) | 0 (0.00%) |  |
| BMI |  |  |  |
| <18.5 kg/m^2^ | 43 (3.96%) | 13 (1.34%) | <0.001* |
| 18.5-23.9 kg/m^2^ | 453 (41.8%) | 328 (33.9%) |  |
| 24.0-27.9 kg/m^2^ | 472 (43.5%) | 500 (51.7%) |  |
| ≥28.0 kg/m^2^ | 117 (10.8%) | 126 (13.0%) |  |
| Smoking |  |  |  |
| Never | 773 (71.2%) | 671 (69.4%) | 0.345 |
| Former | 117 (10.8%) | 98 (10.1%) |  |
| Current | 195 (18.0%) | 198 (20.5%) |  |
| Alcohol drinking |  |  |  |
| Never | 861 (79.4%) | 676 (69.9%) | <0.001* |
| Former | 81 (7.47%) | 70 (7.24%) |  |
| Current | 143 (13.2%) | 221 (22.9%) |  |
| Physical activity |  |  |  |
| Mild | 770 (71.0%) | 665 (68.8%) | 0.196 |
| Moderate | 272 (25.1%) | 248 (25.6%) |  |
| Heavy | 43 (3.96%) | 54 (5.58%) |  |
| Living standard |  |  |  |
| Poor | 48 (4.42%) | 34 (3.52%) | 0.052* |
| Average | 743 (68.5%) | 626 (64.7%) |  |
| Good | 294 (27.1%) | 307 (31.7%) |  |
| Nervousness |  |  |  |
| Mild | 809 (74.6%) | 712 (73.6%) | 0.103 |
| Moderate | 229 (21.1%) | 193 (20.0%) |  |
| Severe | 47 (4.33%) | 62 (6.41%) |  |
| Dyslipidemia |  |  |  |
| No | 892 (82.2%) | 691 (71.5%) | <0.001* |
| Yes | 193 (17.8%) | 276 (28.5%) |  |
| Diabetes |  |  |  |
| No | 899 (82.9%) | 806 (83.4%) | 0.811 |
| Yes | 186 (17.1%) | 161 (16.6%) |  |
| Family history of hypertension |  |  |  |
| No | 482 (44.4%) | 370 (38.3%) | 0.001* |
| Yes | 554 (51.1%) | 570 (58.9%) |  |
| Unknown | 49 (4.52%) | 27 (2.79%) |  |
| Family history of stroke |  |  |  |
| No | 874 (80.6%) | 777 (80.4%) | 0.016* |
| Yes | 171 (15.8%) | 173 (17.9%) |  |
| Unknown | 40 (3.69%) | 17 (1.76%) |  |
| Family history of CHD |  |  |  |
| No | 929 (85.6%) | 841 (87.0%) | 0.004* |
| Yes | 105 (9.68%) | 106 (11.0%) |  |
| Unknown | 51 (4.70%) | 20 (2.07%) |  |

^a^ Low-level exposure group.

^b^ High-level exposure group.

**Table S4.** Stratified analyses of the association between the studied vitamin co-exposure patterns and systolic/diastolic blood pressure.

| **Subgroups** | **Systolic blood pressure** | | | **Diastolic blood pressure** | | |
| --- | --- | --- | --- | --- | --- | --- |
|  | **Adjusted model** |  |  | **Adjusted model** |  |  |
|  | **β coefficient (95% CI)** | ***p* value** | ***p* for interaction** | **β coefficient (95% CI)** | ***p* value** | ***p* for interaction** |
| **Age, years** |  |  |  |  |  |  |
| <65 | 0.003 (-0.003,0.009) | 0.354 |  | **0.008 (0.001,0.014)** | **0.021** |  |
| ≥65 | -0.001 (-0.007,0.006) | 0.87 | 0.75 | 0 (-0.008,0.007) | 0.916 | **0.05** |
| **Sex** |  |  |  |  |  |  |
| Male | 0.003 (-0.003,0.009) | 0.272 |  | 0.004 (-0.003,0.01) | 0.25 |  |
| Female | -0.001 (-0.007,0.006) | 0.803 | 0.718 | 0.001 (-0.006,0.008) | 0.808 | 0.347 |
| **BMI, kg/m^2^** |  |  |  |  |  |  |
| <24 | -0.003 (-0.01,0.004) | 0.456 |  | -0.002 (-0.01,0.006) | 0.701 |  |
| ≥24 | 0.003 (-0.002,0.009) | 0.206 | 0.494 | 0.005 (-0.001,0.011) | 0.128 | 0.216 |
| **Region** |  |  |  |  |  |  |
| North | 0.003 (-0.003,0.009) | 0.338 |  | 0.004 (-0.003,0.01) | 0.245 |  |
| South | -0.002 (-0.008,0.004) | 0.491 | 0.478 | -0.001 (-0.008,0.006) | 0.78 | 0.601 |
| **Education** |  |  |  |  |  |  |
| Primary school or less | -0.003 (-0.009,0.004) | 0.454 |  | -0.005 (-0.013,0.003) | 0.204 |  |
| General intermediate education | 0.007 (-0.001,0.015) | 0.1 |  | **0.01 (0.001,0.019)** | **0.024** |  |
| General secondary education or higher | 0.001 (-0.007,0.009) | 0.774 | 0.174 | 0.006 (-0.003,0.014) | 0.185 | **0.004** |
| **Smoking** |  |  |  |  |  |  |
| Never | 0.001 (-0.004,0.006) | 0.609 |  | 0.002 (-0.004,0.008) | 0.454 |  |
| Former | **0.016 (0.001,0.031)** | **0.037** |  | 0.011 (-0.006,0.028) | 0.21 |  |
| Current | -0.006 (-0.016,0.003) | 0.205 | 0.175 | -0.003 (-0.013,0.008) | 0.633 | 0.792 |
| **Alcohol drinking** |  |  |  |  |  |  |
| Never | -0.001 (-0.006,0.003) | 0.557 |  | 0 (-0.005,0.006) | 0.982 |  |
| Former | 0.012 (-0.007,0.031) | 0.216 |  | 0.005 (-0.018,0.027) | 0.694 |  |
| Current | 0.009 (-0.002,0.019) | 0.113 | 0.242 | 0.008 (-0.003,0.019) | 0.137 | **0.031** |
| **Living standard** |  |  |  |  |  |  |
| Good | 0.001 (-0.007,0.009) | 0.769 |  | 0.004 (-0.005,0.012) | 0.411 |  |
| Average | 0.001 (-0.004,0.006) | 0.664 |  | 0.002 (-0.004,0.008) | 0.542 |  |
| Poor | -0.009 (-0.039,0.022) | 0.57 | 0.800 | -0.013 (-0.049,0.023) | 0.475 | 0.531 |
| **Nervousness** |  |  |  |  |  |  |
| Mild | 0.001 (-0.004,0.006) | 0.634 |  | 0.002 (-0.004,0.007) | 0.54 |  |
| Moderate | 0 (-0.009,0.009) | 0.975 | 0.869 | 0.001 (-0.009,0.012) | 0.782 |  |
| Severe | 0 (-0.024,0.024) | 0.983 | 0.959 | 0.008 (-0.016,0.033) | 0.51 | 0.984 |
| **Season** |  |  |  |  |  |  |
| May 1 to October 30 | 0.003 (-0.002,0.008) | 0.257 |  | 0.003 (-0.003,0.008) | 0.327 |  |
| November 1 to April 30 | -0.001 (-0.009,0.007) | 0.864 | 0.605 | 0.001 (-0.008,0.01) | 0.772 | 0.948 |
| **Use of anti-hypertension drug** |  |  |  |  |  |  |
| No | 0 (-0.007,0.006) | 0.898 |  | 0.002 (-0.005,0.01) | 0.542 |  |
| Yes | 0.002 (-0.004,0.007) | 0.509 | 0.26 | 0.002 (-0.004,0.008) | 0.438 | 0.523 |
| **Dyslipidemia** |  |  |  |  |  |  |
| No | 0.001 (-0.004,0.005) | 0.765 |  | 0.002 (-0.003,0.008) | 0.365 |  |
| Yes | 0.002 (-0.007,0.012) | 0.662 | 0.365 | 0 (-0.01,0.011) | 0.957 | 0.542 |
| **Diabetes** |  |  |  |  |  |  |
| No | 0.001 (-0.003,0.006) | 0.552 |  | **0.005 (0,0.01)** | **0.048** |  |
| Yes | 0 (-0.01,0.011) | 0.957 | 0.995 | **-0.013 (-0.024, -0.001)** | **0.035** | 0.596 |

If not stratified, adjusted for age, sex, BMI, region, physical activity, living standard, nervousness, education level, smoking status, alcohol drinking status, the family history of chronic diseases (hypertension, stroke and coronary heart disease), the comorbidity (dyslipidemia and diabetes) and the use of anti-hypertension drug.

Bold values indicated statistical significance.

**Sampling population 1**:

Individuals with hypertension with complete screening records (physical exam, questionnaire, and biological samples) recruited from 9 provinces* in China by December 2017 (N=6246)

**Sampling population 2**:

Individuals with hypertension with complete screening records (physical exam, questionnaire, and biological samples) recruited from 14 provinces† in China by June 2018 (N=19780‡)

**Sample 1**:

Individuals with hypertension sampling from 9 provinces in China who were enrolled from June to August 2017 (N=800)

**Sample 2**:

Individuals with hypertension sampling from 14 provinces in China who were enrolled from February

2017 to May 2018 (N=1543)

Individuals with hypertension sampling from 14 provinces in China (N=2343)

2052 individuals included

Excluding individuals with outliers (N=291)

Random sampling design stratified by provinces

Random sampling design stratified by provinces, sex and age groups

**Figure S1.** **Flow chart of the study participants.**

* Sample 1 includes 9 provinces in China (Gansu, Liaoning, Beijing, Hebei, Jiangsu, Shanxi, Sichuan, Guangxi and Hunan)

† Sample 2 includes 9 provinces in China (Gansu, Heilongjiang, Liaoning, Shandong, Anhui, Beijing, Hebei, Jiangsu, Ningxia, Yunnan, Shanxi, Sichuan, Guangxi and Hunan)

‡ Individuals in Sampling population 1 were totally included in Sampling population 2, except for those 800 individuals who have been sampled for Sample 1.

**
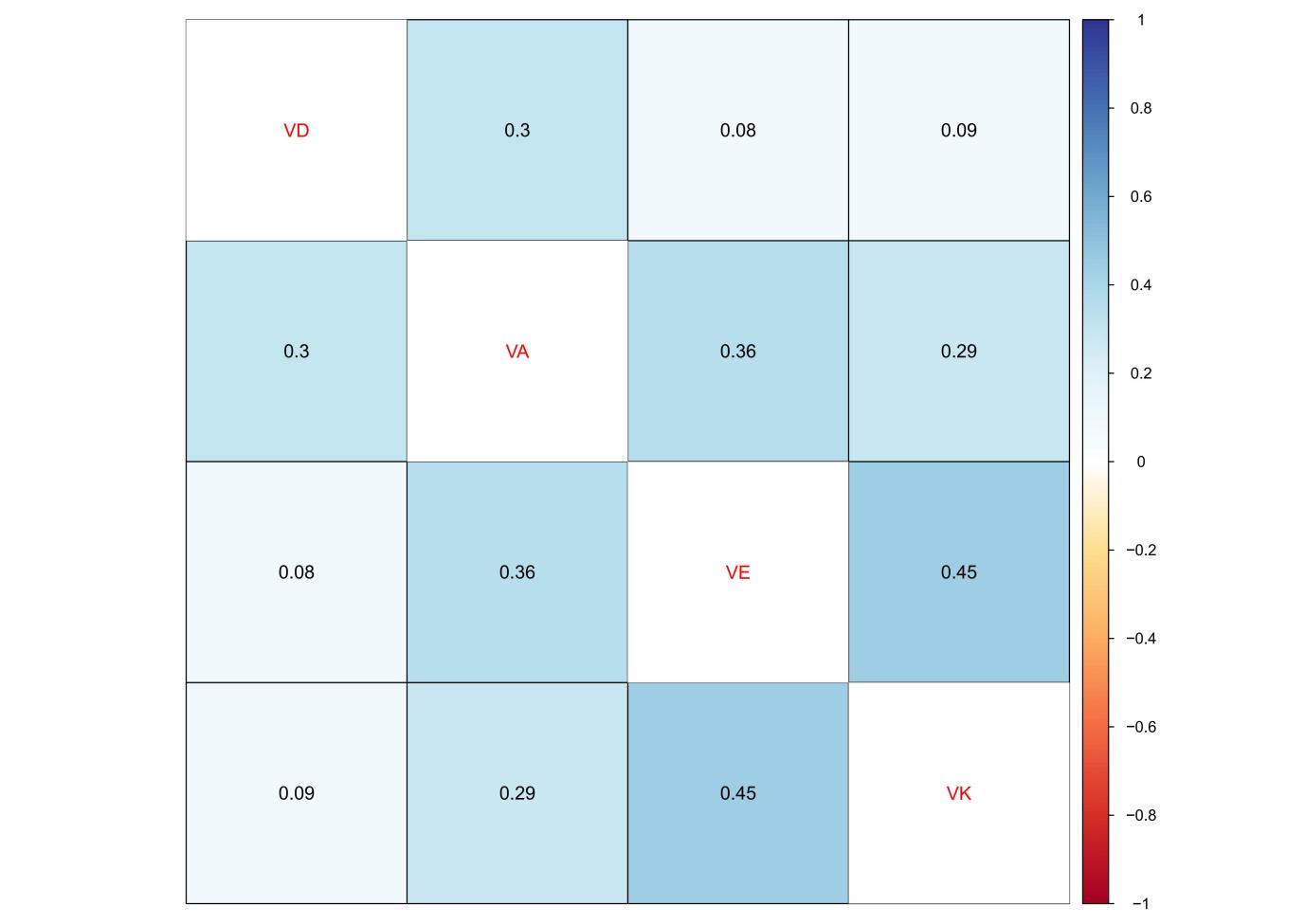
Figure S2. Heatmap elucidation of pairwise correlations of the four lipid-soluble vitamins in plasma.**

VA, vitamin A; VD, vitamin D; VE, vitamin E; VK, vitamin K. All *p*-value for correlation coefficient were significant (*p* < 0.05).


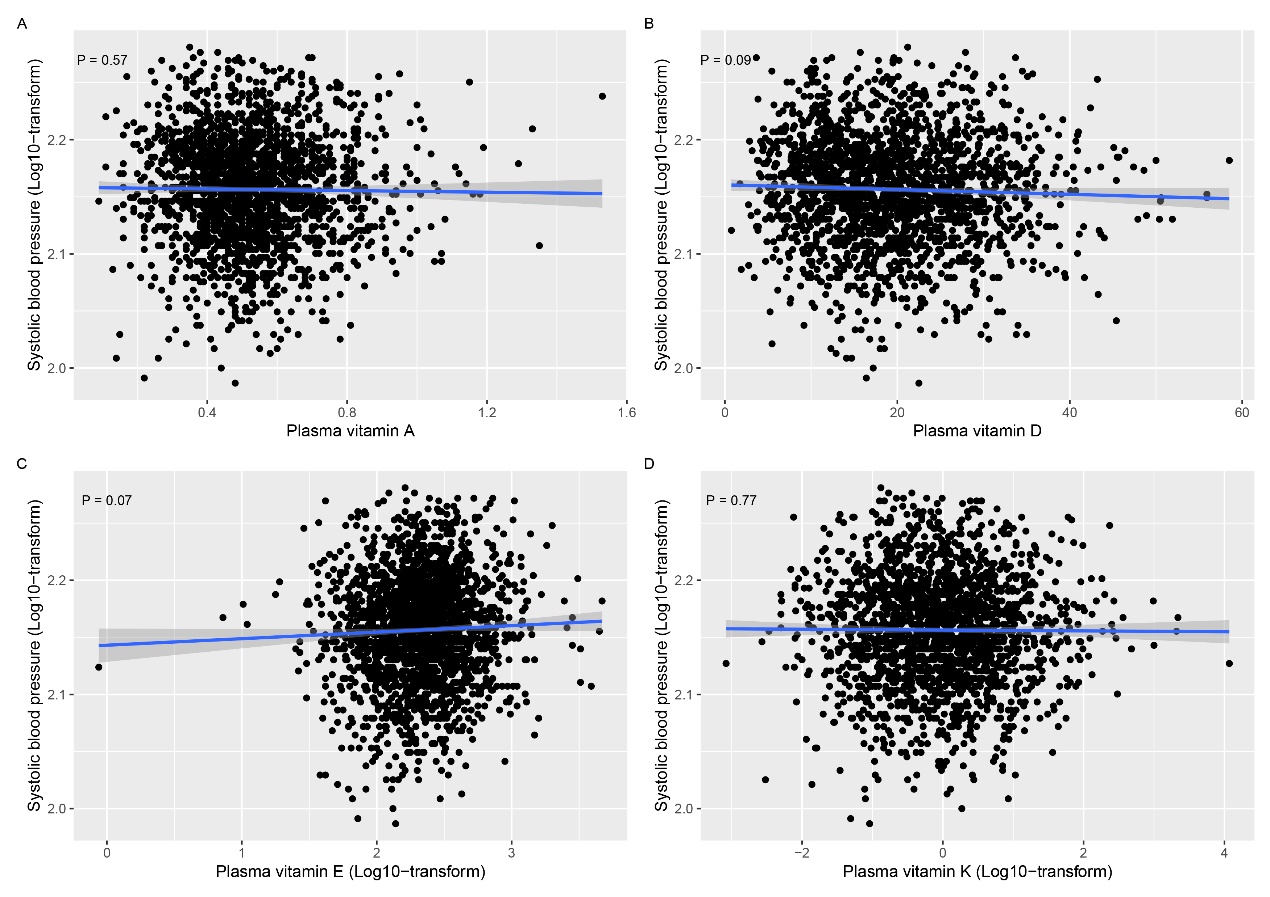


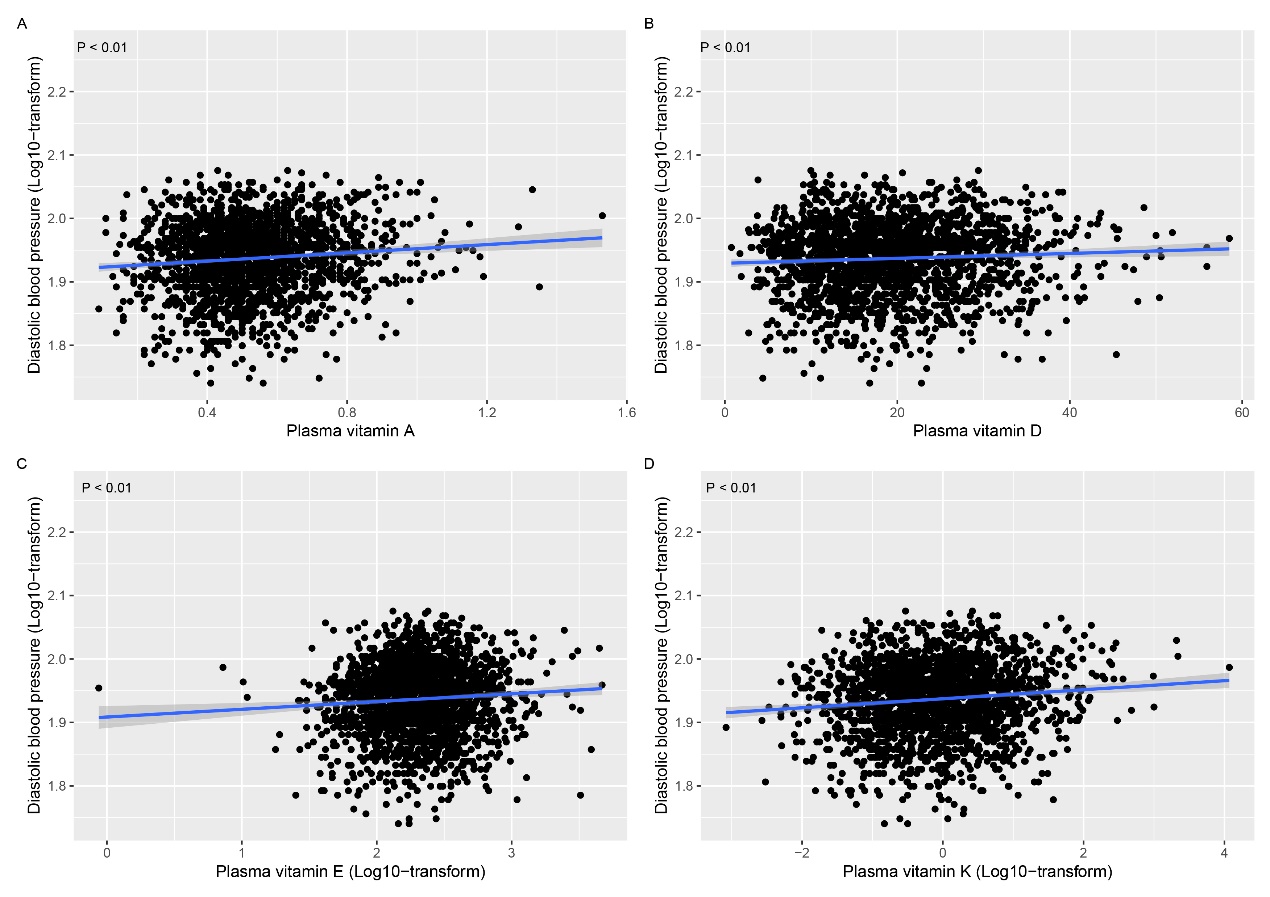


**Figure S3. A scatter plot with a fitted line and 95% CIs showing the association between plasma vitamin concentration and systolic/diastolic blood pressure.**

**
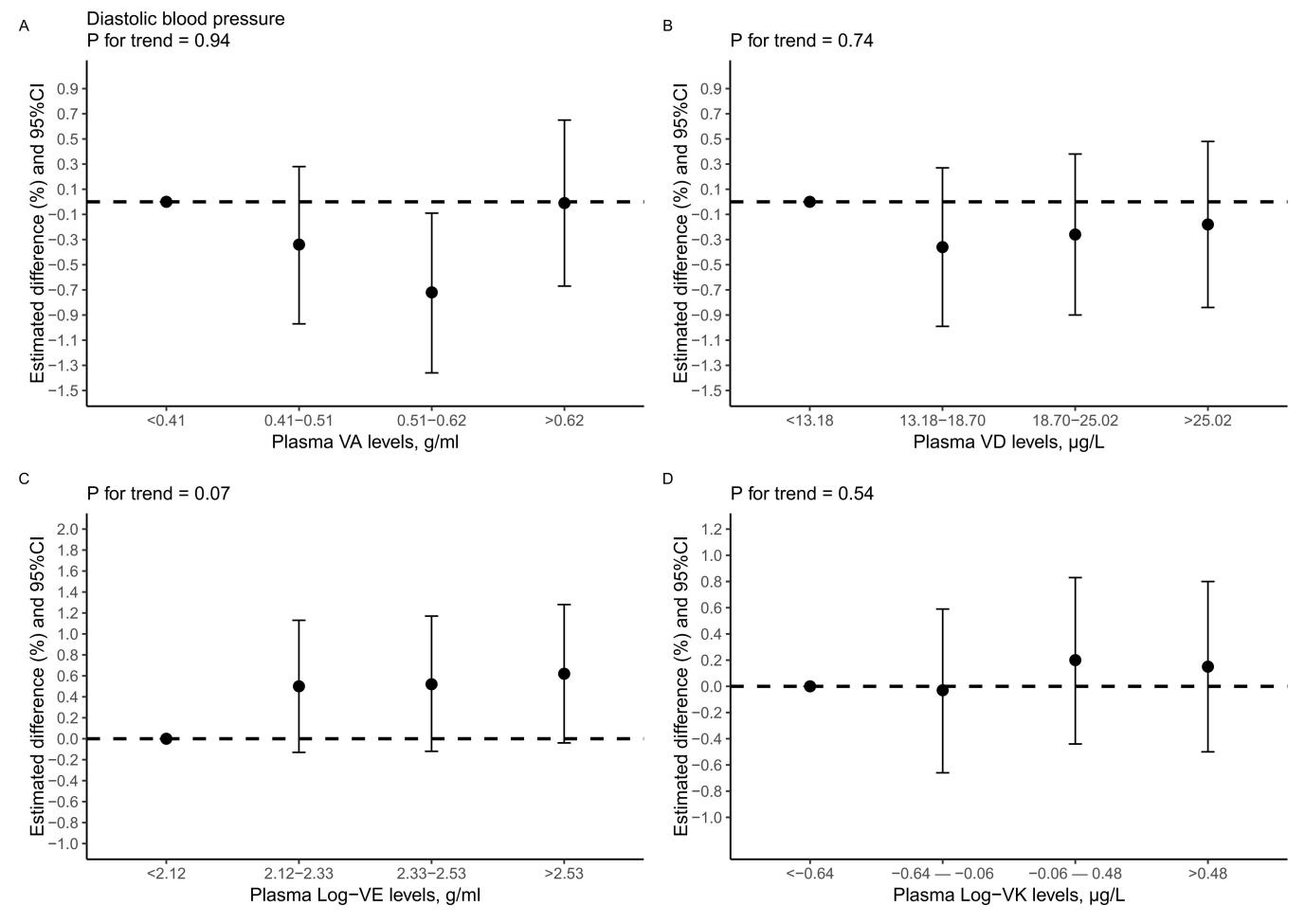
Figure S4. Estimated difference (%) and 95% CI in diastolic blood pressure with *p* for trend for each interquartile in plasma vitamin concentrations.**


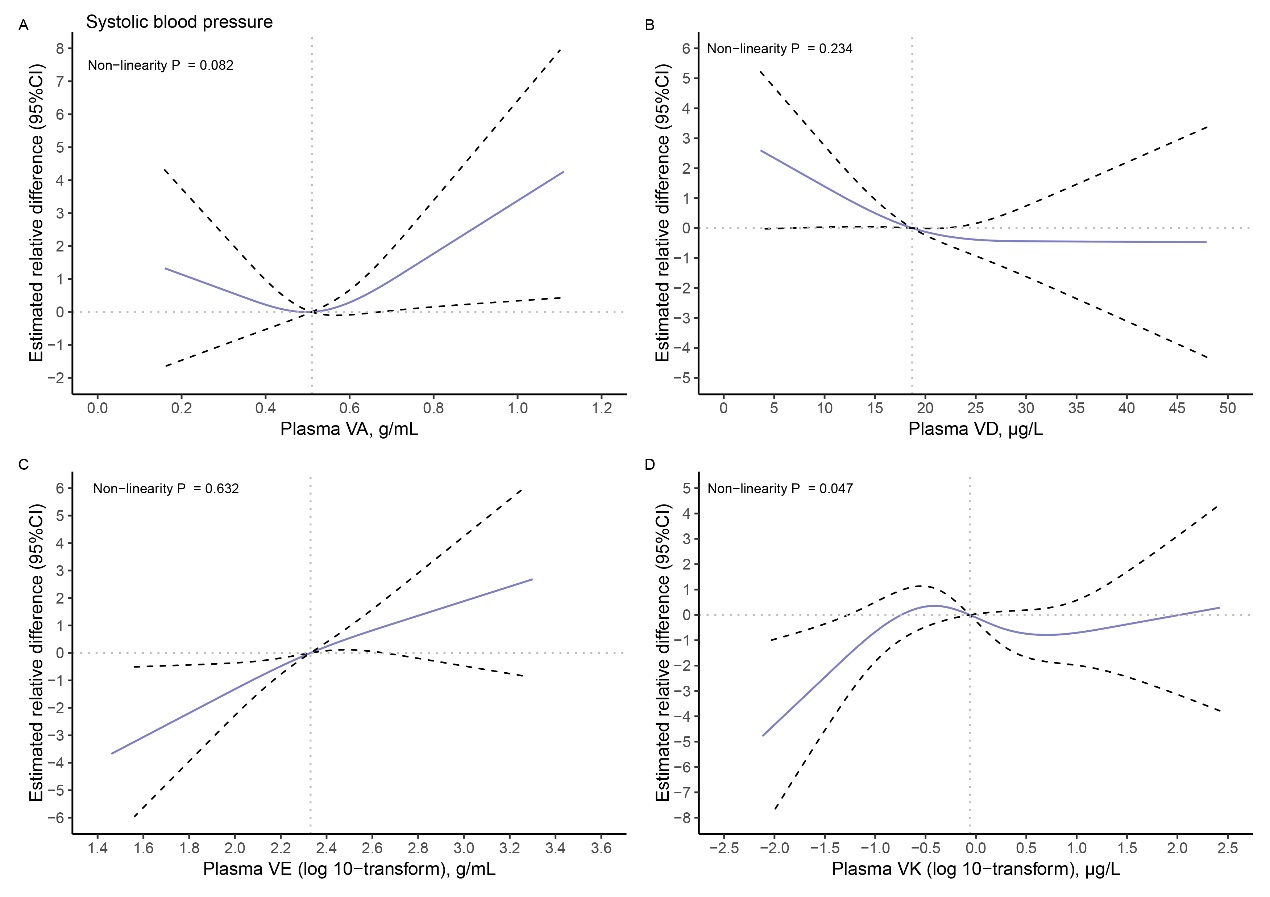

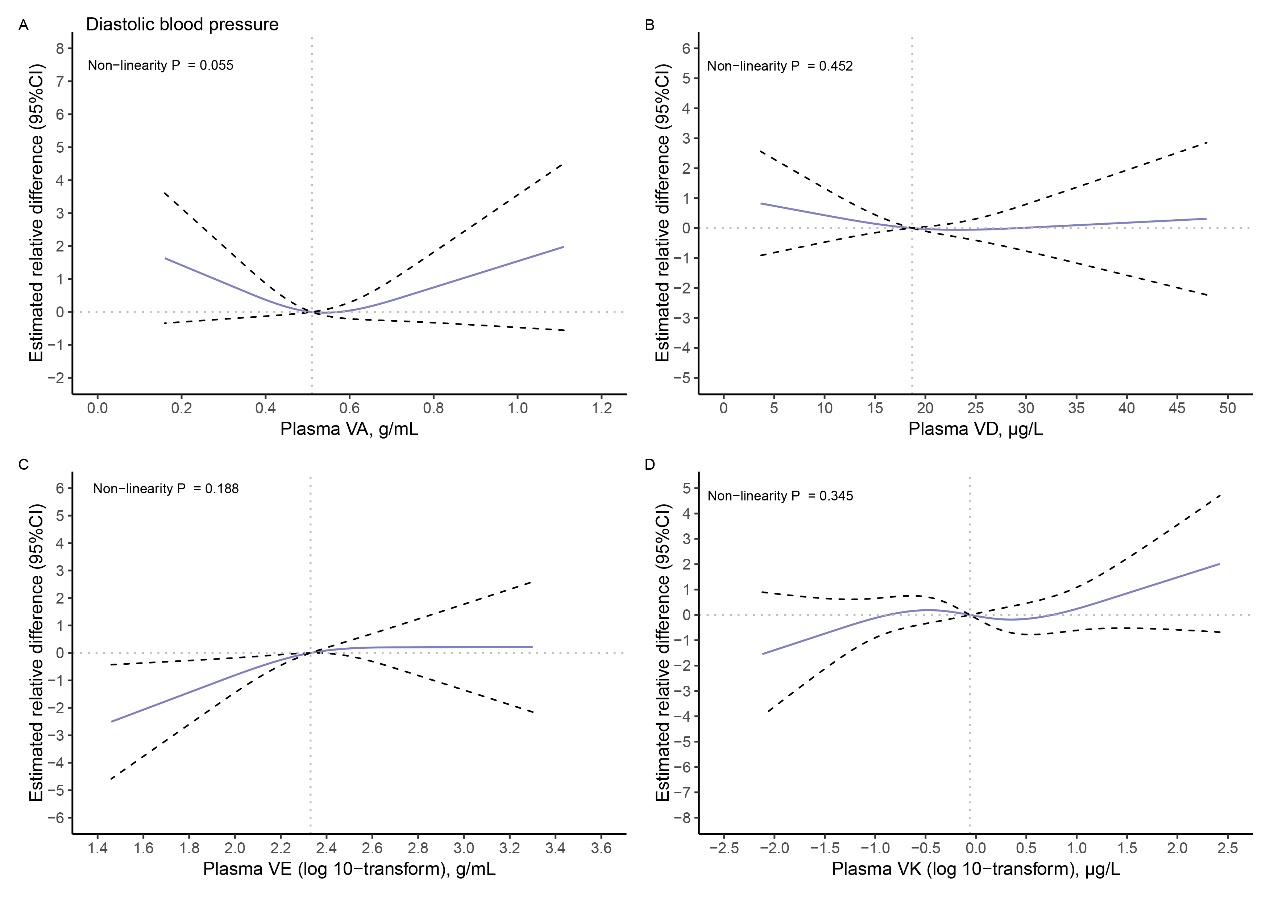


**Figure S5. Detection of non-linear relationship of plasma vitamin concentrations with systolic/diastolic blood pressure according to restricted cubic spline regression models.** The reference value was the blood pressure corresponding to the median of each vitamin.
